# Bibliometric Analysis of the Inflammasome and Pyroptosis in Brain

**DOI:** 10.3389/fphar.2020.626502

**Published:** 2021-01-20

**Authors:** Yuhua Chen, Yan Li, Limin Guo, Jun Hong, Wenjuan Zhao, Ximin Hu, Cuicui Chang, Wei Liu, Kun Xiong

**Affiliations:** ^1^ Central Laboratory of Medicine School, Xi’an Peihua University, Xi’an, China; ^2^ Department of Anatomy and Neurobiology, School of Basic Medical Science, Central South University, Changsha, China; ^3^ Department of Neurosurgery, First Affiliated Hospital of Xiamen University, Xiamen, China; ^4^ Department of Histology and Embryology, School of Basic Medical Science, Xinjiang Medical University, Urumqi, China; ^5^ Clinical Medicine Eight-year Program, 02 Class, 17 Grade, Xiangya School of Medicine, Central South University, Changsha, China; ^6^ Hunan Key Laboratory of Ophthalmology, Changsha, China

**Keywords:** bibliometric analysis, pyroptosis, inflammasome, brain, web of science

## Abstract

**Background:** Considering the pivotal role of inflammasome/pyroptosis in biological function, we visually analyzed the research hotspots of inflammasome/pyroptosis related to the brain in this work through the method of bibliometrics from the Web of Science (WOS) Core database over the past two decades.

**Methods:** Documents were retrieved from WOS Core Collection on October 16, 2020. The search terms and strategies used for the WOS database are as follow: # 1, “pyroptosis”; # 2, “pyroptotic”; # 3, “inflammasome”; # 4, “pyroptosome”; # 5 “brain”; # 6, “# 1” OR “# 2” OR “# 3” OR “# 4”; # 7, “# 5” AND “# 6”. We selected articles and reviews published in English from 2000 to 2020. Visualization analysis and statistical analysis were performed by VOSviewer 1.6.15 and CiteSpace 5.7. R2.

**Results:** 1,222 documents were selected for analysis. In the approximately 20 years since the pyroptosis was first presented, the publications regarding the inflammasome and pyroptosis in brain were presented since 2005. The number of annual publications increased gradually over a decade, which are involved in this work, and will continue to increase in 2020. The most prolific country was China with 523 documents but the United States was with 16,328 citations. The most influential author was Juan Pablo de Rivero Vaccari with 27 documents who worked at the University of Miami. The bibliometric analysis showed that inflammasome/pyroptosis involved a variety of brain cell types (microglia, astrocyte, neuron, etc.), physiological processes, ER stress, mitochondrial function, oxidative stress, and disease (traumatic brain injuries, stroke, Alzheimer’s disease, and Parkinson’s disease).

**Conclusion:** The research of inflammasome/pyroptosis in brain will continue to be the hotspot. We recommend investigating the mechanism of mitochondrial molecules involved in the complex crosstalk of pyroptosis and regulated cell deaths (RCDs) in brain glial cells, which will facilitate the development of effective therapeutic strategies targeting inflammasome/pyroptosis and large-scale clinical trials. Thus, this study presents the trend and characteristic of inflammasome/pyroptosis in brain, which provided a helpful bibliometric analysis for researchers to further studies.

## Introduction

With the rapid development of cell biology and molecular biology in recent decades, the mystery of cell death has been gradually revealed by scholars, which is implicated in various human diseases. Cell death includes accidental cell death (ACD, a biologically uncontrolled process) or regulated cell death (RCD) (Ruan et al., 2019; Tang et al., 2019). ACD is an instantaneous, catastrophic, and uncontrolled biological process, whereas RCD is molecularly defined and finely regulated (Galluzzi et al., 2018; Tang et al., 2019). Cell death may occur in multiple forms in response to calcium disorder, inflammation, free radical production, mitochondrial dysfunction, etc. (Tang et al., 2019). Uncontrolled RCD of a single or mixed type can lead to human diseases, including neurodegeneration, atherosclerosis, autoimmune diseases, and infectious diseases (Fan et al., 2017; Jorgensen et al., 2017; Kolb et al., 2017; Bäck et al., 2019). Since apoptosis was defined by John Kerr, Andrew Wyllie, and Alastair Currie in 1972 (Kerr et al., 1972), about a dozen types of RCD have been identified in succession (Tang et al., 2019), ranging from noninflammatory (e.g., apoptosis and ferroptosis) to highly proinflammatory (e.g., pyroptosis and necroptosis) cell death (Kolb et al., 2017; Galluzzi et al., 2018; Wang et al., 2020b).

In 2001, Cookson and his colleagues first proposed the concept of pyroptosis from the Greek roots pyro, relating to fire or fever, and ptosis (to-sis) to denote a falling, to describe the proinflammatory programmed cell death (PCD) (Cookson and Brennan, 2001), which is lytic and proinflammatory and characterized by the formation of plasma membrane pores that compromise membrane integrity (Vande and Lamkanfi, 2016; Frank and Vince, 2019). Both pyroptosis and apoptosis can be regulated by caspase, but pyroptosis is initiated by inflammatory caspase-1, caspase-4, and caspase-5 (caspase-1/4/5 in humans and caspase-1/11 in mice), instead of the classical apoptotic molecule caspase-3 (Yuan et al., 2016; Jorgensen et al., 2017; Van Opdenbosch and Lamkanfi, 2019). Researches during the past decade have demonstrated that caspase-1 family proteases are expressed as cytoplasmic enzyme and become active after their recruitment by the interactions between conserved protein domains to inflammasomes, cytosolic scaffolds that are assembled by particular pattern recognition receptors (PRRs) (Latz et al., 2013; Man and Kanneganti, 2015; Broz and Dixit, 2016), which is classified as pathogen-associated molecular patterns (PAMPs) and danger-associated molecular patterns (DAMPs) (Vande and Lamkanfi, 2016). Inflammasomes are a group of protein complexes built around several proteins, including NOD-like receptor family pyrin domain-containing 3 (NLRP3) and NLR-family CARD-containing protein 4 (NLRC4), and absent in melanoma 2 (AIM2), NLRP6, and pyrin (Strowig et al., 2012). Different types of inflammasome recruitment and assembly are activated by receiving different stimulation. The NLRP3 inflammasome is activated by a large variety of signals, including PAMPs and DAMPs (Jo et al., 2016; Kelley et al., 2019; Pan et al., 2020), whereas the AIM2 and NLRC4 inflammasomes are activated only by specific PAMPs, double-stranded DNA (dsDNA from bacteria or host cells), and specific bacterial proteins, respectively (Strowig et al., 2012). The NLRP1 inflammasome is also activated by nonpathogen-associated triggers, including extracellular Aβ, intracellular ATP depletion, and intracellular ion flux downstream of the P2X4/7 receptors, or by human disease-associated mutations (Mitchell et al., 2019). Clostridioides difficile toxin B and pertussis toxin induce the pyrin inflammasome activation by breaking the intracellular homeostasis (Dumas et al., 2014), as well as the bile acid analogs (Alimov et al., 2019). When cells suffer from external pathogens or disruption of intracellular equilibrium, PRRs is initiated and then inflammasome sensors recruit procaspase-1 family (with caspase activation and recruitment domain (CARD)) either directly through homotypic binding of CARD or indirectly through a pyrin domain (PYD) using the adaptor apoptosis-associated speck-like protein containing a CARD (ASC) (composition with a PYD and a CARD) (Strowig et al., 2012) that activates caspase-1 family to mediate the formation of cell membrane pores by gasdermins (GSDM) family activation and maturation of interleukin (IL)-1β and IL-18, a major driver of pyroptotic event downstream of inflammasome activation (Shi et al., 2017; Broz et al., 2020).

Inflammasomes involve canonical and noncanonical types that are abnormal activation in the context of pathogen, metabolic imbalances, or tissue damage (Broz and Dixit, 2016; Lu et al., 2019). The canonical caspase-1-dependent inflammasomes are involved in pyrin, NLRP1, NLRP3, NLRC4, and AIM2 (Rathinam and Fitzgerald, 2016) and linked to the release of mature IL-1β and IL-18, while the noncanonical caspase-1-dependent inflammasomes are induced by cytosolic LPS from invading Gram-negative bacteria in macrophages, monocytes, or other cells (Kayagaki et al., 2015; Broz et al., 2020). GSDMD is regarded as the pivotal effector of pyroptosis (Shi et al., 2017; Broz et al., 2020), that is directly cleaved by caspase-1 or caspase-11 through canonical and noncanonical types to produce a 22 kDa°C- (GSDMD-C) and a 3l kDa N-terminal fragment (GSDMD-N) (Kayagaki et al., 2015; Wang et al., 2020a). Further studies have demonstrated that pyroptosis and apoptosis pathways engage in bidirectional crosstalk (Taabazuing et al., 2017), involving apoptotic caspases that participate in lytic cell death and inflammation following apoptotic stimulation via mediating GSDMD (Taabazuing et al., 2017; Chen et al., 2019a) and GSDME (Rogers et al., 2017; Wang et al., 2017; Zheng et al., 2020). GSDMD and pyroptotic activity in apoptotic cells are inactivated by caspase-3/7-dependent cleavage at aspartate D87 (D88 in mice) which inactivates the protein by cleaving within the inactive p20/p10 fragments of GSDMD (Taabazuing et al., 2017). Caspase-3 causes cleavage and activation of GSDME, which induces pyroptosis and loss of membrane integrity that is the conversion from apoptosis (Rogers et al., 2017).

**FIGURE 1:**
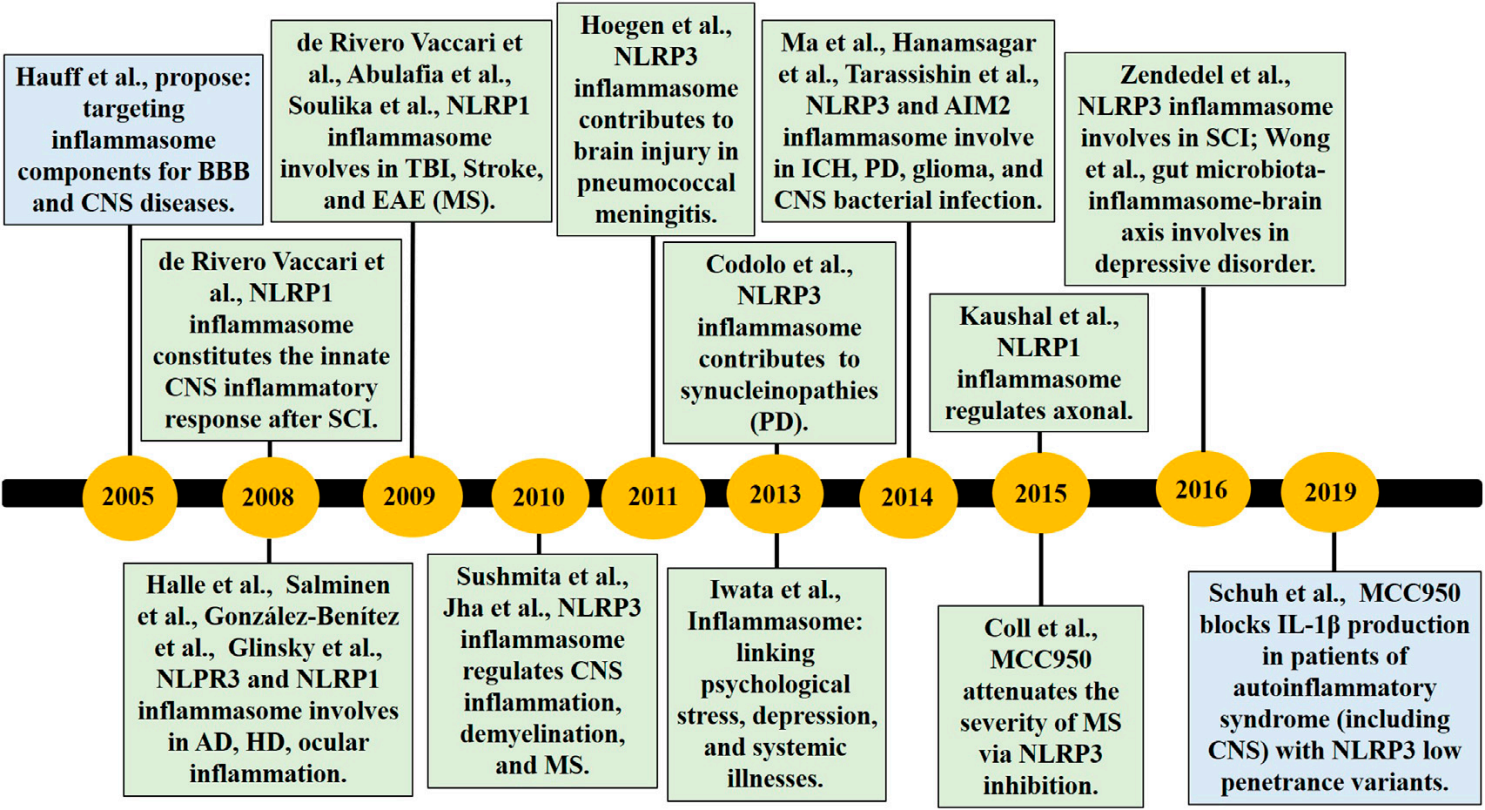
The timeline of part key discoveries in the field of the inflammasome/pyroptosis in CNS diseases. AD, Alzheimer’s disease; BBB, blood-brain barrier; CNS, central nervous system; EAE, experimental autoimmune encephalomyelitis; HD, Huntington’s disease; ICH, Intracerebral Hemorrhage; MS, multiple sclerosis; PD, Parkinson’s disease; SCI, spinal cord injury; TBI, traumatic brain injuries.

Pyroptosis is related to multiple diseases and can be involved in a variety of acute and chronic injuries (Alfonso-Loeches et al., 2014; Israelov et al., 2020; Xu et al., 2019; Zeng et al., 2019). In recent years, studies have found that inflammation is widely found in the various central nervous system (CNS) diseases (Figure 1), and the occurrence and development of traumatic brain injuries (TBI) (de Rivero Vaccari et al., 2009; Liu et al., 2018; Chen et al., 2019c; Irrera et al., 2020), stroke (Abulafia et al., 2009; Poh et al., 2019), Alzheimer’s disease (AD) (Halle et al., 2008; Flores et al., 2018), Parkinson’s disease (PD) (Codolo et al., 2013; Yan et al., 2020), multiple sclerosis (MS) (Soulika et al., 2009; McKenzie et al., 2018), Huntington’s disease (HD) (Glinsky, 2008; Paldino et al., 2020), spinal cord injury (SCI) (de Rivero Vaccari et al., 2008; de Rivero Vaccari et al., 2012; Jiao et al., 2020), and other diseases depend on the inflammasome. Pyroptosis contributes to the pathogenesis of sepsis-associated encephalopathy (SAE), with inhibition of either caspase-1 or NLRP3 improving survival and reducing SAE-associated neurocognitive deficits, neuroinflammation, and GSDMD-mediated pyroptosis in mouse models (Fu et al., 2019; Xu et al., 2019). In the context of direct CNS infection, pathogens activate the NLRP3 inflammasome in neuronal, glial, and myeloid cells (Man et al., 2017; Duan et al., 2020), and the NLRP3 and AIM2 inflammasomes are engaged in a *Staphylococcus aureus* brain abscess model (Hanamsagar et al., 2014). Clinical evidence suggests that patients with TBI are higher cerebrospinal fluid (CSF) levels of the inflammasome proteins NLRP3, AIM2, ASC, and caspase-1 (Adamczak et al., 2012; Adamczak et al., 2014; Wallisch et al., 2017), and CSF NLRP3 levels are increased after TBI in infants and children and independently associated with younger age and poor outcome (Wallisch et al., 2017). Furthermore, NLRP1, NLRP3, NLRC4, and AIM2 inflammasomes have been confirmed to be involved in TBI pathology in animal models, highlighting the multiformity of danger signals resulting from tissue injury (Adamczak et al., 2014; Freeman and Ting, 2016; Irrera et al., 2020). NLRP3 knockout or pharmacological inhibitor is beneficial for the outcomes of TBI animal models through inhibiting pyroptosis and neuroinflammation (Chen et al., 2019c; Chen et al., 2019d; Liu et al., 2018; O’Brien et al., 2020; Swanson et al., 2019), so NLRP3 may be a potentially effective biomarker and therapeutic target for TBI (O’Brien et al., 2020; Swanson et al., 2019). Inflammasome protein ASC is also regarded as the biomarker of TBI and stroke (Kerr et al., 2018a; Kerr et al., 2018b), whereas the physiological function of crosstalk of multi-RCDs by molecules remains to be further determined in normal and disease progression that will provide more sufficient evidence for clinical diagnosis and therapy.

Over the 20 years since pyroptosis is first proposed, the research of inflammasome/pyroptosis in brain has yielded progress, but some issues remain to be explored. How does inflammasome transmit, transform, or cascade inflammatory mediators between glia and neural circuits and ultimately cause neural function alteration? We visually analyzed the research hotspots of inflammasome related to the brain in this work through the method of bibliometrics from the WOS Core database over the past two decades. The data deduce the research trend of the inflammasome in the brain worldwide, which may provide new design ideas for further investigation planning and light up the basic and clinical studies.

## Data and Methods

### Data Collection

We retrieved all literature data regarding the inflammasome/pyroptosis in brain indexed in the Web of Science (WOS) Core Collection (Clarivate Analysis, Boston, United States; http://apps.webofknowledge.com/WOS_GeneralSearch_input.do?product=WOS&SID=7DLaapxWMwSkqZf4LRr&search_mode=GeneralSearch). The term pyroptosis was detected with MeSH (https://www.ncbi.nlm.nih.gov/mesh), whereas the “pyroptosis”, “inflammasome”, and “brain” show other expressions, such as “pyroptotic” and “pyroptosome”. The articles from 2000 to 2020 (October 16, 2020) were searched, the language type was set to English, and the document type was set to article and review.

The search terms and strategies used for the WOS database are as follows: # 1, “pyroptosis”; # 2, “pyroptotic”; # 3, “inflammasome”; # 4, “pyroptosome”; # 5 “brain”; # 6, “# 1” OR “# 2” OR “# 3” OR “# 4”; # 7, “# 5” AND “# 6”.

A total of 14,343 documents of “inflammasome OR pyroptosis” and 1,222 documents of “inflammasome OR pyroptosis AND brain” were retrieved from WOS Core Collection, and then the documents were used to make visual analysis ultimately. The deadline for researched publications was October 16, 2020.

## Methods

WOS-based literature analysis was conducted to understand the general information about the distribution of publication years, journals, organizations, authors, and research fields. The ranking order is the Standard Competition Ranking method. Then, the VOSviewer and CiteSpace were used to perform the bibliometric analysis and network visualization, including the top authors, keywords, research cooperation relationships, and cocitation network analysis of reference. The “citation report” function from Web of Science was applied to assess citation rates and h-index.

We choose the keywords and key references to predict the research prospect and research hotspot. Keywords and key references were analyzed by VOSviewer and CiteSpace. The parameters of the VOSviewer were set as follows: Method (Linlog/modularity). The parameters of CiteSpace were set as follows: Method (LLR), time slicing (2005–2020), years per slice (1), term source (all selection), node type (choose one at a time), and selection criteria (top 50 objects). The silhouette value means the cluster network homogeneity (value >0.7, indicating high reliability). The Q value of the cluster network means the modularity of the network (Q > 0.5, indicating significant network cluster structure).

## Results

### The Tread of Publication and Research

**FIGURE 2:**
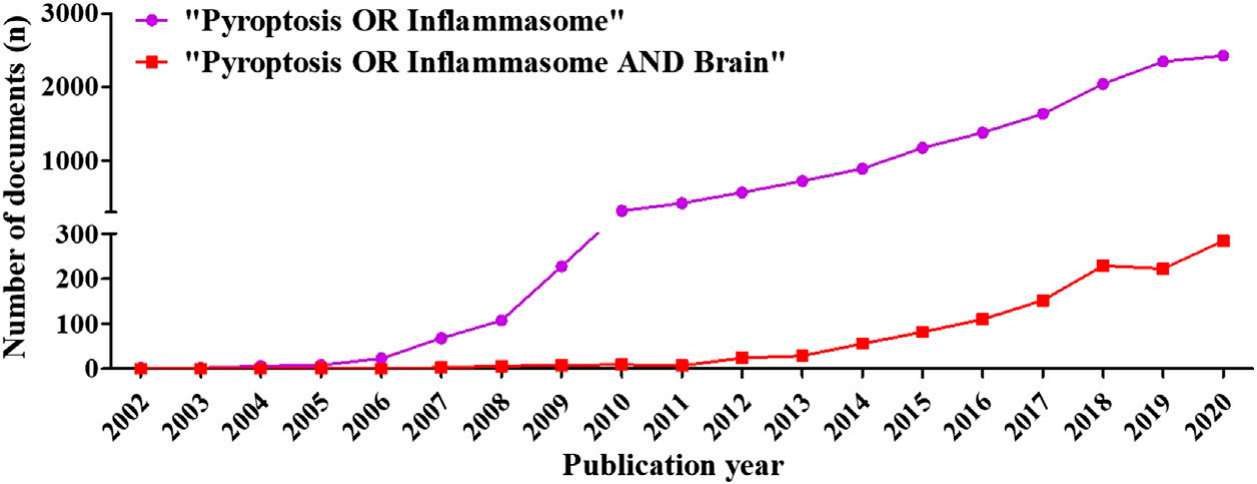
Distribution of publications on “inflammasome OR pyroptosis” and “inflammasome OR pyroptosis AND Brain” according to years. The number of documents was grown relatively slowly from 2002 to 2011, whereas that rose sharply from 2012 to 2020.

There were 11,021 (76.84%) articles and 3,322 (23.16%) reviews among the 14,343 documents of “inflammasome OR pyroptosis” and 954 (78%) articles and 269 (22%) reviews among the 1,222 documents of “inflammasome OR pyroptosis AND brain”. Compared with the review documents of inflammasome/pyroptosis, the reviews of pyroptosis/inflammation in brain presented a high proportion, suggesting that inflammasome/pyroptosis has more possibilities to reveal in the brain. The chronological distribution of published documents was shown in Figure 2. The data showed that the publications of inflammasome/pyroptosis and its role in brain were of sustained growth. From 2012, the growth trend was very fast (Figure 2). We speculated that the number of documents on “inflammasome OR pyroptosis AND brain” and “inflammasome OR pyroptosis” was likely to be about 300 and 2,800 in 2020, respectively. It suggests that inflammasome/pyroptosis attracted more and more attention all over the world, which indicates that inflammasome/pyroptosis in brain will be a continuing hotspot in the future.

### Countries/Regions

**FIGURE 3:**
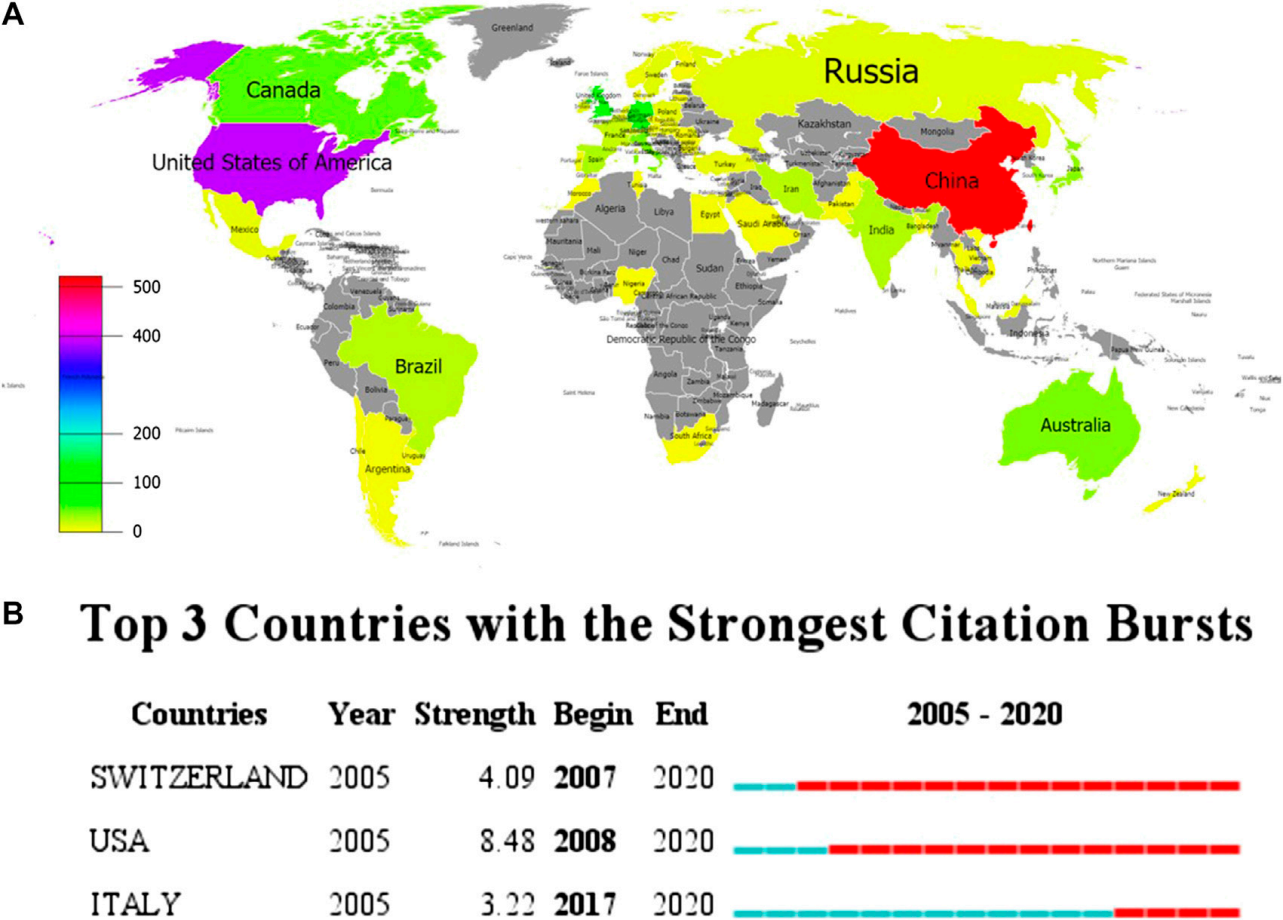
Spatial distribution of global publications. **(A)** The documents of “inflammasome OR pyroptosis AND Brain”. The different colors represented the difference in the number of documents, and the gray represented the countries with no publications. **(B)** Top 10 countries/regions with the strongest citation bursts by CiteSpace. ɣ: 1.0, minimum duration: 1. The strongest citation burst means that a variable has a great change in a short period. Red bars indicate the duration of the burst.

113 countries/regions were involved in the publication of inflammasome/pyroptosis, but just 58 countries/regions dabbled in its role in brain (Figure 3A). According to the statistical analysis, some of the documents were completed in cooperation with multiple countries/regions. United States (392), China 523), and Germany 84) were the top three countries/regions of the documents of inflammasome/pyroptosis and its role in brain (Table 1). The number of documents of inflammasome/pyroptosis in brain in China was more than that in the United States, but its citation and centrality were less, which was 4,450 vs. 5,186. As shown in the top three countries/regions with the strongest citation bursts (a great change of publications in a short period) (Figure 3B), the United States showed the highest burst strength with 8.48 from 2008 to 2020, indicating that there are many scholars studying inflammasome/pyroptosis in brain in the United States from 2008 to 2020. The citation bursts of Switzerland and Italy were 4.09 and 3.33, respectively, but the scholars in Italy showed a later burst from 2017 to 2020. The strong citation bursts of all top three countries/regions were continued until 2020, suggesting that there will still be a large number of scholars joining the field of research.

**TABLE 1:** Top 10 the most productive countries/regions on inflammasome/pyroptosis in brain.

Rank	Country/region	Documents	Citations	Total link strength	Links
1	China	523	8,215	122	25
2	United States	392	16,328	217	37
3	Germany	83	5,641	80	25
4	United Kingdom	72	1,942	84	31
5	Canada	49	1,581	39	17
6	Italy	44	1,121	14	9
7	Australia	41	1,902	48	18
8	Japan	38	921	28	8
9	Republic of Korea	37	882	26	10
10	Spain	29	1,916	39	18

### Organizations

**FIGURE 4:**
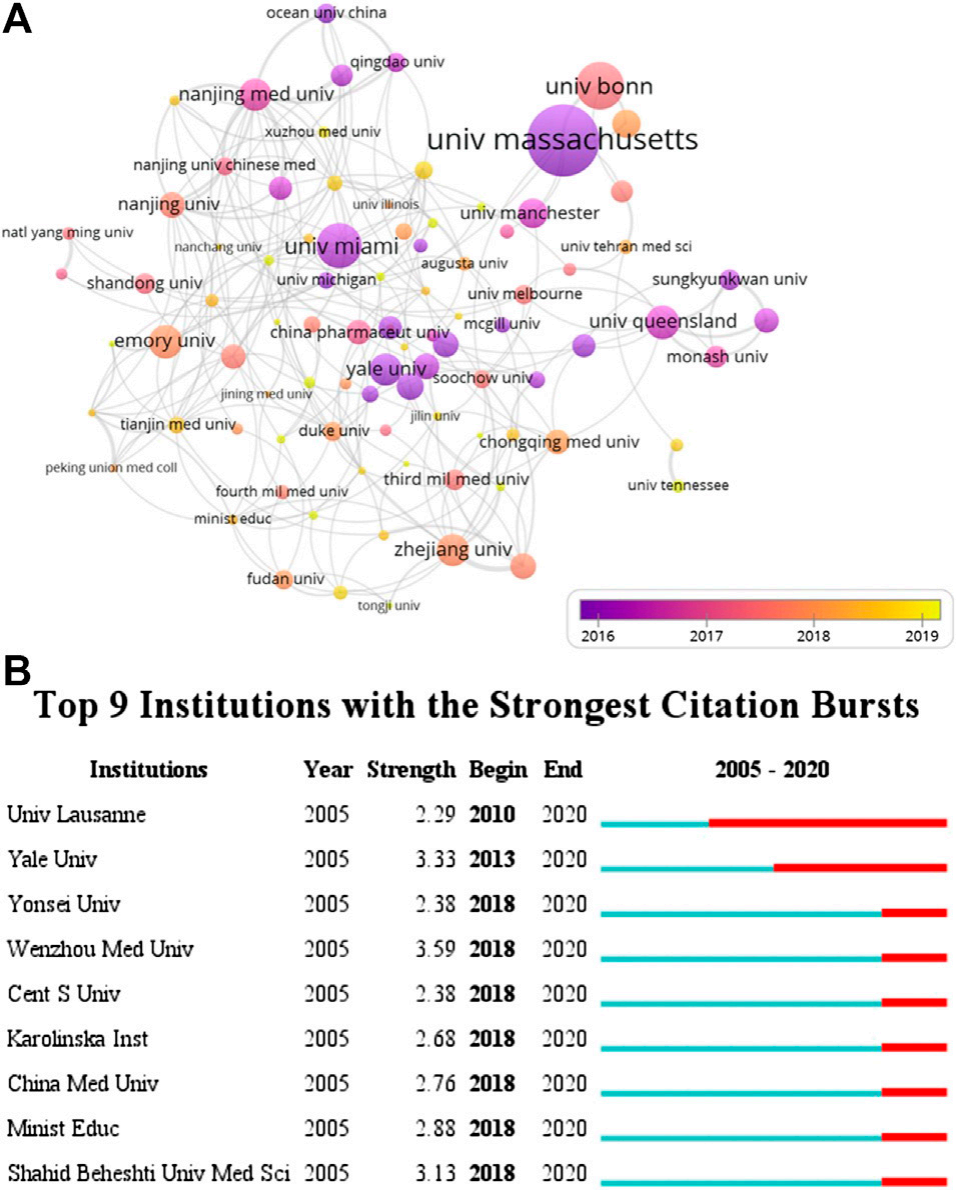
Coauthor analysis of organizations. **(A)** VOSviewer 1.6.14 was used for analysis, the method was Linlog/modularity, weight was citations, and scores were the average published year. The thickness of the lines indicates the strength of the relationship. The color means the average published year. **(B)** Top nine institutions with the strongest citation bursts by CiteSpace. ɣ: 0.8, minimum duration: 2.

According to VOSviewer analysis, 1,222 documents were published by 1,357 different organizations and 117 met the threshold (minimum number of documents of an organization: 5). After excluding disjointed organizations, the remaining 117 organizations were used for the visualization map. Top 10 prolific organizations were listed in Table 2, including six Chinese organizations, three United States organizations, and one England organization. The most prolific organizations were Zhejiang University and University of Miami (both n = 39, 3.19%), and the citations and total link strength (keywords and other keywords: total cooccurrence, including repeat cooccurrence) were 872 and 27 *vs.* 1,707 and 4. The data indicate that the University of Miami has more cooperation with other organizations. Unexpectedly, the total citations of Chinese six organizations (n = 3,695) were lower than those in the University of Massachusetts (n = 4,464). The cooccurrence relations also showed that the University of Massachusetts, University of Bonn, University of Miami, University of Barcelona, and University of Queensland were the top five citations of organizations (Figure 4A). The data suggest that the publications from the organizations of the United States, Germany, and Spain were still dominated in the field of inflammasome/pyroptosis in brain. The yellow node presented that the average published year was 2019; therefore, Sun Yat-sen University was the most recent organization to publish more articles on inflammasome/pyroptosis in brain. It suggests that these might be emerging research organizations in this field. For burst monitoring of institutions (Figure 4B), the top three ranked items were Wenzhou Medical University burst from 2018 to 2020, followed by Yale University burst from 2013 to 2020 and Shahid Beheshti University Medical Science burst from 2018 to 2020.

**TABLE 2:** Top 10 the most productive organizations.

Rank	Organizations	Country	Documents	Citations	Total link strength
1	Zhejiang University	China	39	872	27
2	University of Miami	United States	39	1,707	4
3	Nanjing Medical University	China	33	901	42
4	University Manchester	England	24	751	7
5	Nanjing University	China	23	558	24
6	Sun Yat-sen University	China	23	291	14
7	China Pharmaceut University	China	20	544	10
8	Chongqing Medical University	China	20	529	9
9	University of Massachusetts	United States	19	4,464	16
10	Loma Linda University	United States	18	564	21

### Journals

By the data analysis, the documents of inflammasome/pyroptosis in brain from 2005 to 2020 were mainly distributed in different journals and the top 10 journals were listed in Table 3. The most prolific journal was the Journal of Neuroinflammation with 69 documents. The 2019 impact factor of these journals ranged from 2.74 to 6.633; among them, Brain, Behavior, and Immunity was the highest but PLoS One was the lowest. By the JCR partition analysis, Q1 was 50%, Q2 was 40%, and Q3 was 10% in this ranking. It is helpful to find out the core journal by analyzing the distribution of publications sources. Judging by the number of publications and impact factors, the Journal of Neuroinflammation may be the most popular journal.

**TABLE 3:** Top 10 with the largest number of publications.

Rank	Journals	Documents	2019 impact factor	2019 JCR partition
1	Journal of Neuroinflammation	69	5.793	Q1
2	Brain, Behavior, and Immunity	36	6.633	Q1
3	Molecular Neurobiology	30	4.5	Q1
4	International Immunopharmacology	25	3.943	Q2
5	Frontiers in Cellular Neuroscience	22	3.921	Q2
6	Frontiers in Immunology	19	5.085	Q1
7	International Journal of Molecular Sciences	19	4.556	Q1
8	Biochemical and Biophysical Research Communications	19	2.985	Q3
9	Frontiers in Neuroscience	17	3.707	Q2
10	PLoS One	17	2.74	Q2

### Authors

It is beneficial for probing the distribution of documents by analyzing core authors. The evaluation criteria of core authors include the number of published documents, the total citations, and h-index. 6,808 authors were involved in the field of inflammasome/pyroptosis in brain with 1,222 publications. The top 10 core authors in this field were listed from 2005 to 2020 in Table 4. de Rivero Vaccari and his colleagues Keane and Dietrich from the University of Miami were ranked in the top three documents. de Rivero Vaccari was the first with 27 documents and his total citations were 1,545, but Latz from the University of Bonn had the highest total citations (n = 3,284) with 10 documents (Table 4). Dietrich and Latz were with 72 h-index and 70 h-index, respectively (Table 4). But Sarlus was with 4 h-index, which might be the result of his low publications. It indicates that the researches of Latz in the field of inflammasome/pyroptosis in brain have been recognized by more scholars, although the team of de Rivero Vaccari has more documents.

**TABLE 4:** Core authors on inflammasome/pyroptosis in brain.

Authors	Organizations	Documents	Citations	h-index
de Rivero Vaccari	University of Miami	27	1,545	27
Keane	University of Miami	22	1,316	34
Dietrich	University of Miami	21	1,425	72
Brough	University of Manchester	15	522	34
Beyer	RWTH Aachen University	14	433	48
Sarlus	University of Bonn	12	2,049	4
Zhang	Loma Linda University	11	473	62
Latz	University of Bonn	10	3,284	70
Chen	Zhejiang University	10	493	26
Zhang	Zhejiang University	10	266	24

**FIGURE 5:**
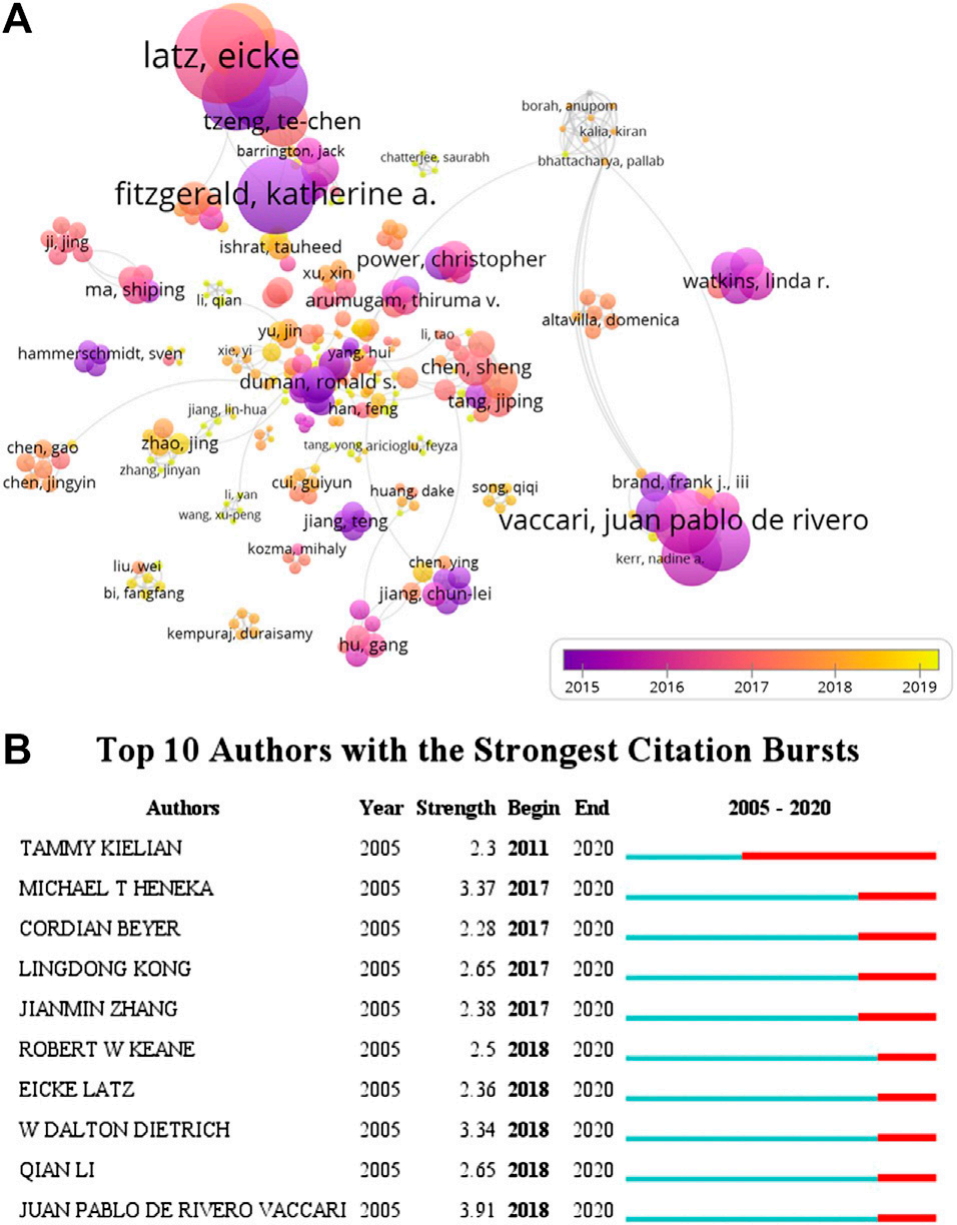
Coauthorship analysis of authors. **(A)** The analysis method was Linlog/modularity in VOSviewer, the weight was citations, and scores were the average published year. The thickness of the lines indicates the strength of the relationship. The color means the average published year. **(B)** Top 10 institutions with the strongest citation bursts by CiteSpace. ɣ: 0.8, minimum duration: 2.

As shown in Figure 5A, the overlay visualization showed the coauthorship relations of authors, including 322 authors. From the visualization map, Latz, Heneka, and Golenbock (University of Massachusetts) were collaborated closely (Figure 5A), indicating that they had close cooperation in this field, and their average published year was in 2015 and 2016, respectively. The data indicated that Latz, Heneka, and Golenbock had been deep in the field for a long time. Furthermore, several emerging researchers (yellow dots) and groups had also begun to converge on the field of inflammasome/pyroptosis in brain (Figure 5A), suggesting that it is still a hotspot. For burst monitoring of authors (Figure 5B), the top three ranked items were de Rivero Vaccari burst from 2018 to 2020, followed by Dietrich burst from 2018 to 2020 and Heneka burst from 2017 to 2020.

### Keywords

**FIGURE 6:**
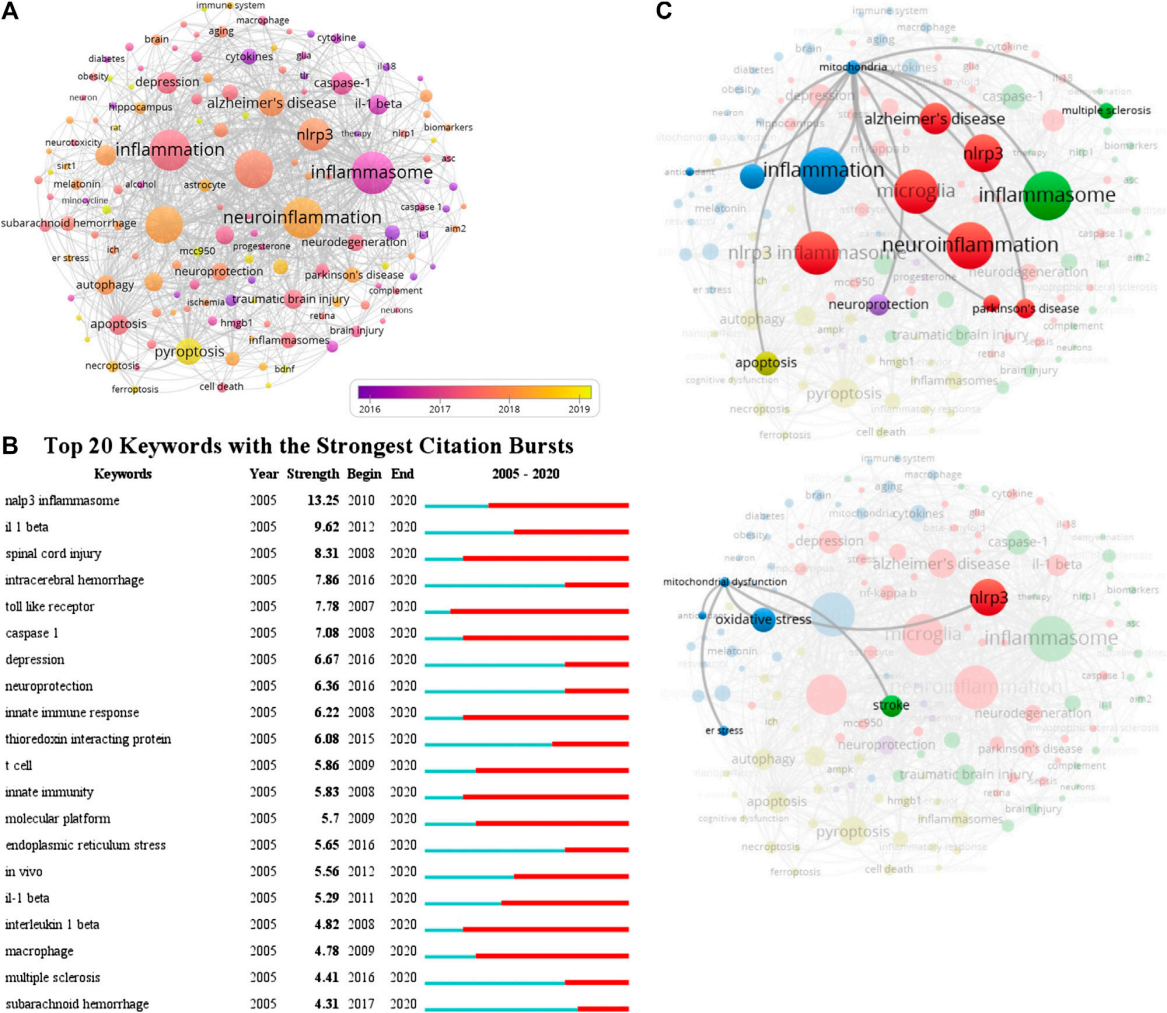
Cooccurrence analysis of author keywords. **(A)** The analysis method was Linlog/modularity in VOSviewer, the weight was an occurrence, and scores were the average published year. The thickness of the lines indicates the strength of the relationship. The color means the average published year. **(B)** Top 20 keywords with the strongest citation bursts by CiteSpace. ɣ: 1.0; minimum duration: 2. **(C)** The relationship between mitochondria and other author keywords was conducted by the network visualization.

A total of 2,271 author keywords were involved in 1,222 documents and 145 met the threshold (minimum number of documents of a keyword: 5). The network visualization map showed the cooccurrence relations of keywords (Figure 6A). The size of the circle indicates occurrences of keywords. The inflammasome, neuroinflammation, inflammation, microglia, NLRP3 inflammasome, NLRP3, and pyroptosis were high-frequency keywords, the average published year of inflammasome was 2016, and then the other keywords were used sequentially from 2017 to 2018 in this field. According to statistical analysis of the author keywords, numerous molecules participated in the progress of inflammasome/pyroptosis activation in microglia, astrocyte, neuron, and other cell types and were involved in the processes of ER stress, mitochondrial dysfunction, and oxidative stress (Figure 6A). At first, these keywords were mostly associated with cerebral ischemia, SCI, and innate immunity in 2016, but now there was growing evidence that they were linked to neurodegeneration, AD, PD, TBI, and neuroprotection (Figure 6A). Recently, the relationship between pyroptosis and other RCDs (such as necroptosis, apoptosis, autophagy, ferroptosis, etc.) was focused on by some scholars (Figure 6A). Figure 6B showed the top 20 keywords with the strongest citation bursts. NLRP3 inflammasome showed the highest burst strength with 13.25 (Figure 6B). The researches of NLRP3 inflammasome busted in 2010 and continued to 2020 (Figure 6B), suggesting that NLRP3 inflammasome is a hotspot. The keywords of caspase-1 (including caspase one and caspase one activation) and IL-1β were also with high strongest citation bursts (Figure 6B). By the network visualization, the relationship between mitochondria and other author keywords showed that mitochondrial researches on the field of inflammasome/pyroptosis in brain remained to be further strengthened (Figure 6C). The data show that NLRP3 inflammasome is a hotspot and many scholars have devoted it to this field.

### Citations

**FIGURE 7:**
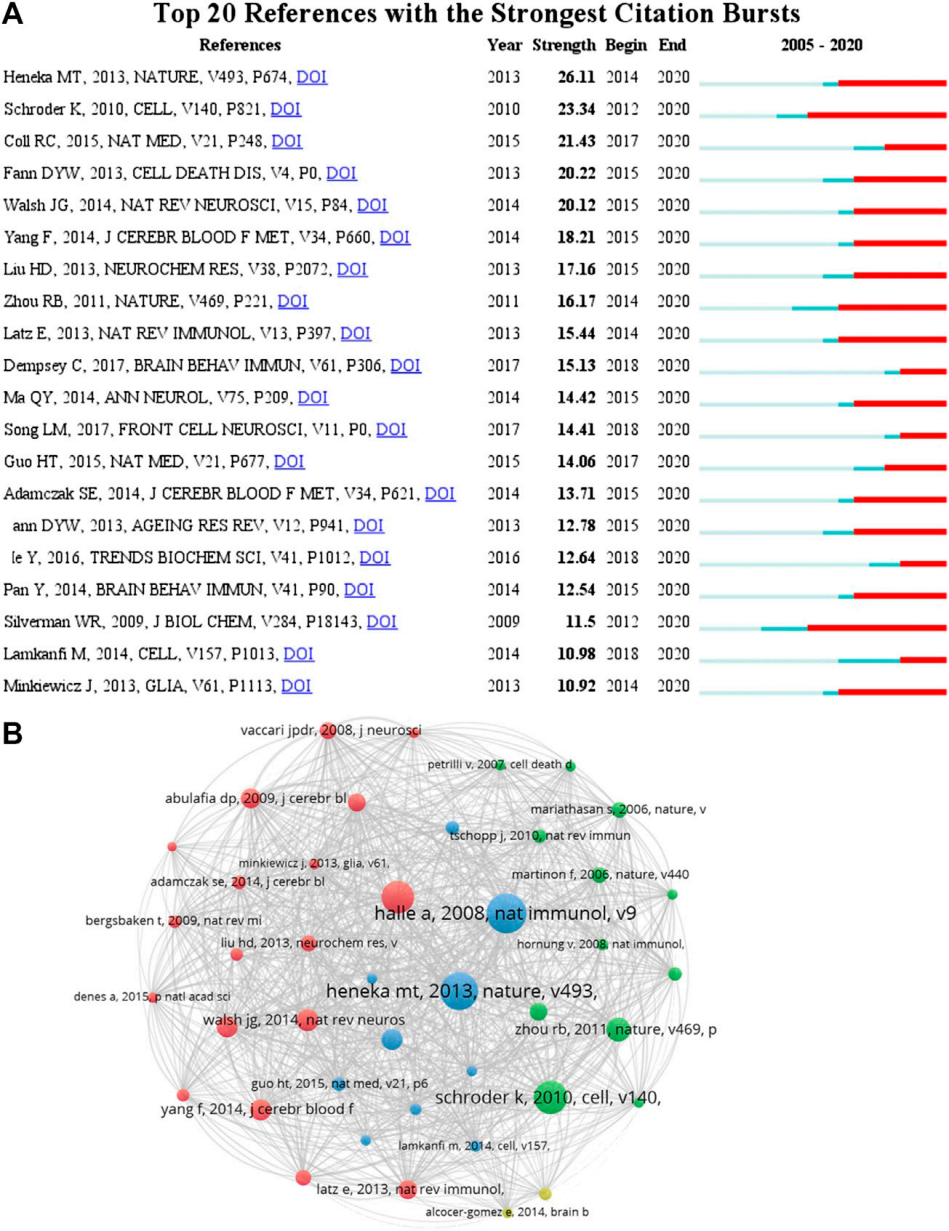
Cocitation analysis of references. **(A)** Top 20 references with the strongest citation bursts by CiteSpace. ɣ: 1.0; minimum duration: 2. The pale color indicates the time range of statistics since 2005, the dark cyan color indicates the time range from publication to the strongest citation bursts, and the red color indicates the duration of the strongest citation bursts. **(B)** The analysis method was Linlog/modularity in VOSviewer, and the weight was citations. The thickness of the lines indicates the strength of the relationship. The purple part of the circle indicated the centrality of documents.

According to the citation analysis of documents, which reflected the citations of documents, the top 10 high citations were listed in Table 5, and the range of citations was from 217 to 1,294. “The NALP3 inflammasome is involved in the innate immune response to amyloid-β” (2018) and had 1,294 citations; “NLRP3 is activated in Alzheimer”s disease and contributes to pathology in APP/PS1 mice”, published by M.T. Heneka et al. (2013), which was with 961 citations; and the third was 889 citations, “The role of inflammation in depression: from evolutionary imperative to modern treatment target” (2016) (Table 5). “Extensive innate immune gene activation accompanies brain aging, increasing vulnerability to cognitive decline and neurodegeneration: a microarray study” was with 217 citations and ranked the last among this ranking (Table 5). Five of those high citations documents focused on the role of inflammasome in AD (published in 2008, 2009, 2012, 2013, and 20,014), which might be an important reason for 84 occurrences of the keywords of AD (with the average published year 2018) and hotspot of AD (Table 5). The top 20 references with the strongest citation bursts were presented (Figure 7A). The article published by Heneka et al. (2013) showed the highest bursts with 26.11 from 2014 to 2020, and the article published by Minkiewicz (2013) showed the lowest bursts with 10.92 from 2014 to 2020 (Figure 7A). The data suggest that a large number of references burst in 2014 and have continued to 2020.

**TABLE 5:** Top 10 cocitation analysis of documents on inflammasome/pyroptosis in brain.

Rank	Title	First author	Source	Publication year	Total citations
1	The NALP3 inflammasome is involved in the innate immune response to amyloid-β	Halle et al. (2008)	Nature Immunology	2008	1,294
2	NLRP3 is activated in Alzheimer’s disease and contributes to pathology in APP/PS1 mice	Heneka et al. (2013)	Nature	2013	961
3	The role of inflammation in depression: From evolutionary imperative to modern treatment target	Miller and Raison (2016)	Nature Reviews Immunology	2016	889
4	Oxidized mitochondrial DNA activates the NLRP3 inflammasome during apoptosis	Shimada et al. (2012)	Immunity	2012	850
5	Innate immune activation in neurodegenerative disease	Heneka et al. (2014)	Nature Reviews Immunology	2014	560
6	Inflammasomes in the CNS	Walsh et al. (2014)	Nature Reviews Neuroscience	2014	301
7	Canonical Nlrp3 inflammasome links Systemic low-Grade inflammation to functional decline in aging	Youm et al. (2013)	Cell Metabolism	2013	246
8	The inflammasome: Pathways linking psychological stress, depression, and systemic illnesses	Iwata et al. (2013)	Brain, Behavior, and Immunity	2013	234
9	Inflammation in Alzheimer’s disease: Amyloid-β oligomers trigger innate immunity defense via pattern recognition receptors	Salminen et al. (2009)	Progress in Neurobiology	2009	226
10	Extensive innate immune gene activation accompanies brain aging, increasing vulnerability to cognitive decline and neurodegeneration: a Microarray study	Cribbs et al. (2012)	Journal of Neuroinflammation	2012	217

To analyze the citations of documents, cocitation analysis of cited references was performed (Figure 7B and Table 6). There were a total of 59,007 cited references; the top high cited references were Halle (Halle et al., 2008), Heneka (Heneka et al., 2013), Schroder (Schroder and Tschopp, 2010), Martinon (Martinon et al., 2002), Zhou (Zhou et al., 2011), Walsh (Walsh et al., 2014), Fann (Fann et al., 2013), Coll (Coll et al., 2015), Yang (Yang et al., 2014), and Abulafia (Abulafia et al., 2009) (Figure 7). The centrality of Halle et al. (2008) and Heneka et al. (2013) was the top two in this ranking, which was consistent with the previous citation analysis of cited reference in Table 6, indicating that these two articles were the most acknowledged documents of inflammasome/pyroptosis in AD.

**TABLE 6:** Top 10 cocitation of cited reference on inflammasome/pyroptosis in brain.

Rank	Title	First author	Source	Publication year	Total citations
1	The NALP3 inflammasome is involved in the innate immune response to amyloid-β	Halle et al. (2008)	Nature Immunology	2008	197
2	NLRP3 is activated in Alzheimer’s disease and contributes to pathology in APP/PS1 mice	Heneka et al. (2013)	Nature	2013	189
3	The inflammasomes	Schroder and Tschopp (2010)	Cell	2010	165
4	The inflammasome: a Molecular platform triggering activation of inflammatory caspases and processing of proIL-beta	Martinon et al. (2002)	Mol. Cell	2002	161
5	A role for mitochondria in NLRP3 inflammasome activation	Zhou et al. (2011)	Nature	2011	120
6	Inflammasomes in the CNS	Walsh et al. (2014)	Nature Reviews Neuroscience	2014	109
7	Intravenous immunoglobulin suppresses NLRP1 and NLRP3 inflammasome-mediated neuronal death in ischemic stroke	Fann et al. (2013)	Cell Death Dis.	2013	108
8	A small-molecule inhibitor of the NLRP3 inflammasome for the treatment of inflammatory diseases	Coll et al. (2015)	Nat. Med.	2015	107
9	NLRP3 deficiency ameliorates neurovascular damage in experimental ischemic stroke	Yang et al. (2014)	J. Cereb. Blood Flow Metab.	2014	105
10	Inhibition of the inflammasome complex reduces the inflammatory response after thromboembolic stroke in mice	Abulafia et al. (2009)	J. Cereb Blood Flow Metab.	2009	99

## Discussion

It has been more than a decade since the proposition of the inflammasome/pyroptosis in the brain (Hauff et al., 2005; Glinsky, 2008; Halle et al., 2008; Abulafia et al., 2009; de Rivero Vaccari et al., 2008; Soulika et al., 2009). The number of annual publications increases gradually over the 15 years involved in this work and will continue to increase in 2020. From the growth curve and burst of keyword, we speculate that more and more researchers are interested in inflammasome/pyroptosis, which remains a research hotspot, and the literature related to inflammasome/pyroptosis will continue to increase in the next decade. According to the article types analysis and the theory of bibliometrics, the majority of articles, but few reviews, predict that more researches will be conducted (Paunkov et al., 2019). Currently, the countries, organizations, authors, and journals of dominants of inflammasome/pyroptosis in brain are most in the Western countries.

According to document analysis, the groups such as Golenbock team, Latz team, Heneka team, and de Rivero Vaccari team, still lead this field. Golenbock and his collaborators are working on inflammasome and AD and advance the research in the process of AD pathogenesis (Heneka et al., 2013; Venegas et al., 2017; Ising et al., 2019). Heneka et al. demonstrate that NLRP3^−/−^ or Casp1^−/−^ mice carrying mutations associated with familial AD are largely protected from loss of spatial memory and other sequelae associated with AD and demonstrated reduced brain caspase-1 and IL-1β activation as well as enhanced Aβ clearance (Heneka et al., 2013). NLRP3 inflammasome deficiency skews microglial cells to an M2 phenotype and results in the decreased deposition of Aβ in the APP/PS1 model of AD (Heneka et al., 2013). They further verify that NLRP3 inflammasome function reduces tau hyperphosphorylation and aggregation by regulating tau kinases and phosphatases and intracerebral injection of fibrillar Aβ-containing brain homogenates induces tau pathology in an NLRP3-dependent manner (Ising et al., 2019). They illustrate the Aβ-cascade hypothesis in AD that neurofibrillary tangles develop downstream of Aβ-induced microglial activation, which involves NLRP3 inflammasome activation in the pathogenesis of tauopathies (Heneka et al., 2013; Ising et al., 2019). de Rivero Vaccari and his collaborators find a lot of problems in their clinical work and demonstrate that inflammasomes are potential biomarkers of TBI and multiple sclerosis, which are correlation with intracranial pressure and outcome (de Rivero Vaccari et al., 2009; Adamczak et al., 2014; Kerr et al., 2018b; Keane et al., 2018; Lee et al., 2019; Pérez-Bárcena et al., 2020). Based on their researches, our group has carried out a series of studies on pyroptosis in TBI since 2016. The data show that downregulation of NRLP3/caspase-1 axis in mice can improve the TBI induced never injury; *in vitro* experiments further confirm that pyroptosis is one of the important forms of cortical neuron death, which is mediated by NLRP3 inflammasome (Liu et al., 2018; Chen et al., 2019c; Chen et al., 2019d). Many emerging scholars are devoting to inflammasome/pyroptosis in brain, but the literature analysis shows that they lack communication and cooperation with the international teams at present, which is like an islet. In the future, more exchanges and cooperation are necessary to promote researches in this field.

In the past two decades, there has been a renaissance in neuroimmunology research in neuroscience. At present, glial cells are still a poorly studied cell population related to neurons (Bassett et al., 2020). Keyword analysis suggests that a variety of brain cell types (including microglia, astrocyte, and neuron) undergo inflammasome/pyroptosis activation when they suffer from ER stress, mitochondrial dysfunction, and oxidative stress (Heneka et al., 2018). Microglia are the prominent innate immune cell in the brain for inflammasome activation (Voet et al., 2019). The typical IL-1β and inflammasome signaling in immunosenescent microglia may herald the neurodegenerative conditions, such as AD and PD (Heneka et al., 2018). Incipiently, Gustin et al. demonstrate that NLRP3 inflammasome is expressed and functional in mouse brain microglia but not in astrocytes (Gustin et al., 2015). With further research, scholars have confirmed that the existence of NLRP3 inflammasome activation is in astrocytes (Freeman et al., 2017; Ebrahimi et al., 2018). NLRC4 and NLRP3 inflammasome activation, as important actors, are involved in lysophosphatidylcholine-induced astrogliosis and microgliosis of multiple sclerosis (Freeman et al., 2017). Ebrahimi et al. show that Aβ_1-42_ triggers NLRP3-inflammasome activation in primary murine astrocytes (Ebrahimi), inducing oligomerization and proinflammatory protease recruitment, which is associated with AD pathology. Some scholars believe that the most interesting questions are about how glia regulate neural circuits, how glia affect behavior, and how glia affect the function of the brain as a highly evolved multicellular organ, not just a collection of neural circuits (Bassett et al., 2020). The most pressing issue in dealing with neuroimmunology is how microglia contribute to CNS disease and how to modulate this contribution and holds the promise of shedding new light on disease mechanisms as well as brain-body and neuroimmune interactions (Bassett et al., 2020). Thus, how does inflammasome transmit, transform, or cascade inflammatory mediators between glia and neural circuits and ultimately cause neural function alteration may be an interesting idea in brain/CNS diseases.

Inflammasome is not only involved in brain diseases but also involved in other multiple diseases, including sepsis, cardiovascular disease, liver diseases, and acute myeloid leukemia (Chen et al., 2019b; Guo et al., 2019; Israelov et al., 2020; Johnson et al., 2018; Zeng et al., 2019). The mechanism and function of inflammasome in other diseases also have important implications for the study of that in the brain. Recently, the relationship between pyroptosis and apoptosis or autophagy is concerned by some scholars (Jabir et al., 2015; Rogers et al., 2017; Taabazuing et al., 2017). GSDMD and pyroptotic activity in apoptotic macrophages are inactivated by caspase-3/7-dependent cleavage at aspartate D87 (Taabazuing et al., 2017), caspase-3 causes pyroptosis by the cleavage and activation of GSDME (Rogers et al., 2017), and GSDME-N targets the mitochondria to release death proteins (Cyt c and HtrA2/Omi) (Rogers et al., 2019). Furthermore, Rogers et al. also demonstrate that inflammasome-generated GSDMD-N can also permeabilize the mitochondria linking inflammasome activation to downstream activation of the caspase-3 (Rogers et al., 2019). Jabir et al. have found that autophagy downregulates *P. aeruginosa* that induces NLRC4 inflammasome activation *via* mitochondrial damage and release of mitochondrial DNA (mtDNA) triggered by the bacterial T3SS (Jabir et al., 2015). The oxidized mtDNA (Shimada et al., 2012) and the new synthesis of mtDNA (Zhong et al., 2018) have been verified to directly induce NLRP3 inflammasome activation during apoptosis. In severe fever with thrombocytopenia syndrome (SFTS) virus infection, the cytosolic mtDNA binds and triggers NLRP3 inflammasome activation resulting in SFTS disease progression and fatal outcome (Li et al., 2020). The mtROS/inflammasome pathway involves arsenic-induced hepatic insulin resistance (Jia et al., 2020) and ozone-induced chronic lung inflammation and emphysema (Wiegman et al., 2020). Researches demonstrate that rapamycin reduces NLRP3 inflammasome activation by inhibiting the mTOR/NF-κB pathway in macrophages (Dai et al., 2019), and mTOR regulates NLRP3 inflammasome activation via reactive oxygen species in murine lupus (Li et al., 2018). Chen et al. have demonstrated that rapamycin-induced mitophagy further enhances the neuroprotection of inhibition of NLRP3 inflammasome activation by MCC950 following TBI (Chen et al., 2019d); this may be crosstalk with mtDNA and/or mtROS. MCC950 as an NLRP3 specificity inhibitor (Coll et al., 2019; Tapia-Abellán et al., 2019), the keywords in this bibliometric study, inhibits NLRP3 inflammasome activation and pyroptosis in a variety of disease models and improves disease process (Coll et al., 2015; Dempsey et al., 2017; Ren et al., 2020; Xu et al., 2020), but that does not alter wound healing in obese mice (Lee et al., 2018). A clinical trial involving 19 autoinflammatory syndrome patients reveals some key findings (Schuh et al., 2019): 1) peripheral blood mononuclear cells from patients with NLRP3 low penetrance variants are more likely to release NLRP3-specific IL-1β, which is demonstrated by inhibition of NLRP3 with MCC950; 2) these patients present NLRP3-independent release of IL-6 and TNF-α; and 3) patients with NLRP3 low penetrance variants may present with severe CNS manifestations and partially respond to IL-1 targeting therapies. These data demonstrate that MCC950 targeting NLRP3 is clinically safe and is a potential treatment strategy for the autoinflammatory syndrome, but the pathological mechanism of NLRP3 involvement in the CNS disease still needs to be confirmed by more clinical samples. Researches provide a conceptual advance in understanding the function of pyroptosis, apoptosis, autophagy, and mitochondria in the program cell death. However, more evidence is still lacking on which regulatory switch molecules directly participate in mtDNA and/or mtROS induced inflammasome activation. It is promising to investigate the mechanism of mitochondrial molecules involved in the complex crosstalk of inflammasome and RCDs in brain glial cells, which will facilitate the development of effective therapeutic strategies targeting inflammasome and large-scale clinical trials.

## Limitations

This is the first bibliometric analysis of inflammasome and pyroptosis in brain, but some limitations should be addressed. Firstly, the deadline for researched publications was October 16, 2020, but WOS Core Collection would also keep updating, some of the documents have been already online in 2021, and this part was omitted in this work. All data in this work could not fully reflect the reality of 2020 and could be a reference. Secondly, the terms of “pyroptosis”, “pyroptotic”, “pyroptosome”, “inflammasome”, “brain”, “English”, “Article”, and “Reviews” were selected that only appeared in the title, abstract, and keywords, but the related terms in the text were not retrieved and analyzed, due to the technical defects. Only articles and reviews were used so no commentaries, patents, abstracts, and thesis were used for this analysis. Furthermore, the document type labels assigned by WOS may be inaccurate. Thirdly, since each published article is limited to only 3 to 10 keywords, some core words in these articles are not included in the bibliometric analysis, and the analysis results may also be affected by the incomplete extraction of keywords. Fourthly, because the search was limited to WOS Core Collection indexed journal, a few documents that were not included by WOS Core Collection were missed. However, we think that this work still can be applied to present the overall situation and general trend for this field.

## Conclusion

This bibliometric study was the first to analyze publications of inflammasome and pyroptosis in brain around the world. The documents are increased year by year, especially since 2012. This field has also unceasingly stimulated a large number of scholars’ interest and research, which will continue to be the hotspot during the next decade. Based on the researches of some Western scholars, it opens the door to the study of the role of inflammasome/pyroptosis role in brain diseases. However, the upstream molecular mechanisms and the role of inflammasome/pyroptosis in brain and disease, especially in clinical samples, remain to be elucidated. Moreover, the precise molecular mechanism of the crosstalk of inflammasome and other RCDs in mitochondrial will be a hotspot, which is an effective approach to illuminate the pivotal neuroprotection of inflammasome/pyroptosis in brain injury. It is promising to investigate the mechanism of mitochondrial molecules involved in the complex crosstalk of inflammasome and RCDs in brain glial cells, which will facilitate the development of effective therapeutic strategies targeting inflammasome and large-scale clinical trials. Thus, this study presents the trend and characteristic of inflammasome/pyroptosis in brain, which provided a helpful bibliometric analysis for researchers to further studies.

## Data Availability Statement

The original contributions presented in the study are included in the article/Supplementary Material, and further inquiries can be directed to the corresponding authors.

## Author Contributions

YC and YL contributed to conceptualization. LG and JH contributed to data collection and verification. XH contributed to the methodology. YC, WZ, CC, and KX contributed to data analysis. YC contributed to writing the original draft. WL and KX contributed to writing, review, and editing.

## Funding

This study was supported by the National Natural Science Foundation of China (nos. 81772134, 81971891, 81901270, and 82072229), Key Research and Development Program of Hunan Province (2018SK2091), and Wu Jie-Ping Medical Foundation of the Minister of Health of China (no. 320.6750.14118).

## Conflict of Interest

The authors declare that the research was conducted in the absence of any commercial or financial relationships that could be construed as a potential conflict of interest.
